# A Variational Autoencoder Cascade Generative Adversarial Network for Scalable 3D Object Generation and Reconstruction

**DOI:** 10.3390/s24030751

**Published:** 2024-01-24

**Authors:** Min-Su Yu, Tae-Won Jung, Dai-Yeol Yun, Chi-Gon Hwang, Sea-Young Park, Soon-Chul Kwon, Kye-Dong Jung

**Affiliations:** 1 Department of Smart Convergence, Kwangwoon University, Seoul 01897, Republic of Koreaksc0226@kw.ac.kr (S.-C.K.); 2 Department of Immersive Content Convergence, Kwangwoon University, Seoul 01897, Republic of Korea; 3 Institute of Information Technology, Kwangwoon University, Seoul 01897, Republic of Korea; 4 Ingenium College of Liberal Arts, Kwangwoon University, Seoul 01897, Republic of Korea

**Keywords:** variational autoencoder, generative adversarial network, progressive neural network, generation, reconstruction, voxel

## Abstract

Generative Adversarial Networks (GANs) for 3D volume generation and reconstruction, such as shape generation, visualization, automated design, real-time simulation, and research applications, are receiving increased amounts of attention in various fields. However, challenges such as limited training data, high computational costs, and mode collapse issues persist. We propose combining a Variational Autoencoder (VAE) and a GAN to uncover enhanced 3D structures and introduce a stable and scalable progressive growth approach for generating and reconstructing intricate voxel-based 3D shapes. The cascade-structured network involves a generator and discriminator, starting with small voxel sizes and incrementally adding layers, while subsequently supervising the discriminator with ground-truth labels in each newly added layer to model a broader voxel space. Our method enhances the convergence speed and improves the quality of the generated 3D models through stable growth, thereby facilitating an accurate representation of intricate voxel-level details. Through comparative experiments with existing methods, we demonstrate the effectiveness of our approach in evaluating voxel quality, variations, and diversity. The generated models exhibit improved accuracy in 3D evaluation metrics and visual quality, making them valuable across various fields, including virtual reality, the metaverse, and gaming.

## 1. Introduction

Rapid advancements in 3D data acquisition and modeling technologies have enabled the effective retrieval of high-quality 3D data from the real world or its generation using user-friendly modeling software [1]. Additionally, recent developments in internet tools, particularly online repositories, have enabled the sharing of 3D shapes among users [2]. Methods utilizing Generative Adversarial Networks (GANs) for generating 3D models capture intricate details, high-level structures, and realistic variations in 3D objects or scenes, creating diverse and visually appealing 3D content that opens up possibilities in entertainment, gaming, metaverses, virtual/augmented reality, computer graphics, and other fields. Understanding the 3D geometry of real-world objects is crucial in many areas of the metaverse and game engineering.

Currently, notable approaches include autoregressive models, Variational Autoencoders (VAEs), and GANs [3,4,5]. Methods such as autoregressive models generate sharp images but suffer from slow evaluation speeds and potential limitations because they directly model conditional distributions over pixels [6,7]. While VAEs are easier to train, their constrained model structure can produce blurry results; however, recent research has improved this aspect [8]. GANs generate sharp images; however, despite recent advancements, they still face challenges, such as having a lower resolution, limitations in handling variations, and training instability [9].

Hybrid methods combine the strengths of these three approaches; however, currently, they cannot match GANs in terms of their image quality [10]. Moreover, generating large 3D volumes becomes challenging owing to gradient issues that are amplified when distinguishing the generated volumes from training data [11]. Furthermore, large volumes require smaller mini-batches owing to memory constraints, further compromising training stability [12].

The VAE latent space not only acts as a low-dimensional continuous space but also poses as a bottleneck. It is typically modeled as a multivariate Gaussian distribution with mean and variance. During training, the VAE aims to reconstruct the input 3D data accurately while regularizing the latent space to adhere to the desired distribution (e.g., a unit Gaussian). It samples the learned latent space to generate new 3D objects or scenes similar to the training data, balancing the reconstruction accuracy and latent space exploration for controlled and diverse 3D generation. Effectively combining VAEs and GANs leverages efficient learning of the latent space and the benefits of controlled features, better reconstruction errors, etc., offering the potential for generating diverse and realistic 3D volumes with improved quality and stability.

Our study highlights a progressive neural network approach similar to that used in the study by Rusu, A.A. et al. [13]. The progressive growth method starts with smaller resolutions, making it easier to gradually expand the generator and discriminator as the training progresses and adding new layers to introduce fine details. This approach significantly accelerates training and enhances stability at higher resolutions. Additionally, residual blocks and shortcut connections are employed using Residual Networks (ResNets), a stable network architecture that alleviates gradient vanishing issues [14].

We focused on the challenges associated with stable and improved 3D object generation and reconstruction. The proposed method, the VAE Cascade GAN, leverages recent developments in volumetric convolution networks and GANs to create a framework for the probabilistic generation of 3D objects in a spatial domain. We robustly combined VAEs and GANs through a structured collection of 3D models that exclusively operated with a variant objective function for the discriminator, creating connections between the 3D generator and the 3D discriminator.

The proposed VAE in our network is a variation of the ResNets that enhances the learning of latent structures in images. The Cascade GAN utilizes the Wasserstein loss with a gradient penalty (GP) to mitigate mode collapse, creating a more stable network structure. By designing the generator and discriminator in a mirrored structure, layers were progressively added to expand the 3D volume scale. During training, the generator layer weights were fine-tuned via a progressive fine-tuning method, whereas the discriminator grew incrementally to larger-volume scales, generating 3D shapes under the supervision of voxel labels. The proposed method offers efficient learning of the latent space and separation of features in data-scarce situations, providing benefits such as better reconstruction errors.

## 2. Related Works

### 2.1. Wasserstein Distance and Gradient Penalty

The 3D objective of a GAN during the training process was carried out to enable the generator to produce data that resembled a real data distribution. The aim was to minimize the difference between the real and generated data distributions. Traditional GANs use metrics such as the Jensen–Shannon divergence or Kullback–Leibler divergence to measure this difference in distribution. However, these metrics have several limitations. When the distributions do not overlap significantly (i.e., easily differentiable distributions), the gradients can point in arbitrary directions [15]. Originally, the Jensen–Shannon divergence was used as a distance metric, but more stable alternatives, including the Wasserstein distance, with improved formulations, such as least squares, marginally augmented absolute deviation, and GP, have been proposed recently [16,17,18,19].

The Wasserstein distance and GP are used to enhance the stability and quality of the GAN training process. The Wasserstein distance measures the “costs” incurred along paths between distributions and serves as a distance measure. This mitigates the mode collapse issue of conventional GANs and promotes stable learning. The GP introduces constraints on the gradients of the discriminator in the boundary region, thereby enabling the learning of more intricate decision boundaries between generated and real data distributions. This alleviates mode collapse problems and enhances training stability.

The Wasserstein GAN algorithm aims to rectify these flaws by minimizing the Wasserstein distance between distributions [20]. This metric is defined for all joint distribution sets $\prod \left(P_{r},P_{q}\right)$ where the marginal distributions are $P_{r}$ and $P_{q}$, as defined in Equation (1):

(1) WPr,Pq=infφ∈∏(Pr,Pq)Ex,y~φ[||x−y||]

The Wasserstein GAN training system is more desirable for enforcing the Lipschitz constraints. Unlike weight clipping, which links the gradients of a differentiable function using a constant, the 1-Lipschitz function penalizes the gradients of the discriminator if they exceed one. Consequently, the following loss function (Equation (2)) was generated for the discriminator:

(2) $$E_{\hat{x}\sim P_{q}}\left[D\left(\hat{x}\right)\right]-{E_{x}}_{\sim P_{r}}\left[D\left(x\right)\right]+\lambda E_{\hat{x}\sim P_{x}}[{\left({\left||\nabla_{\hat{x}}D(\hat{x})|\right|}_{2}-1\right)}^{2}]$$

The 3D VAE GAN is a deep learning-based generative model that combines VAEs and GANs to generate data in the form of 3D volumes [21]. The VAE is a variant of an autoencoder that encodes input data into a low-dimensional latent variable and decodes it to generate outputs that closely resemble the original inputs. The VAE models latent variables as probability distributions and generates data by incorporating noise during sampling. A GAN consists of a generator and a discriminator that learn through competition. The generator generates fake data, whereas the discriminator distinguishes between fake and real data.

### 2.2. Generative Modeling Using VAEs

Previous studies on 3D model structures and generators have included voxel grids, point clouds, meshes, implicit functions, etc. Voxel-based methods represent 3D objects using voxel grids, where each voxel represents a small 3D unit. Generative models, such as the 3D VAE or 3D GAN, can help understand the voxel grid distribution and generate new 3D models. The latent variables in a VAE represent the semantic features of the data, allowing for the control and manipulation of specific features of the generated volumes.

The 3D GAN, an extension of the 2D image generation approach using GANs, pioneered unsupervised 3D voxel-based generation [22]. The 3D GAN generates a 64 × 64 × 64 resolution from the sampled random noise. Its generator is designed based on unsupervised methods [23]. The cross-entropy loss used in traditional GAN models adapts to the discriminator, whereas the generator’s main goal is to synthesize shapes as it adapts to the 2D domain.

The VAE is utilized to encode parametric primitives into the latent space for sampling, connecting primitive GANs and 3D GANs. This model can be jointly trained across all categories in the dataset, and the generated results can be enhanced by introducing the 3D-VAE-IWGAN, which employs a dense layer instead of the sigmoid layers in the 3D GAN [24]. Another approach introduced a voxel-based VAE for comprehending 3D models. The encoder comprises a convolution architecture with a dense layer, and the decoder has a similar but inverted structure. To improve the performance, they introduced Voxception-ResNet, which was inspired by the ResNets [25].

Another study introduced structural awareness loss within the VAE-based framework using a pre-trained structural decoder to enhance generation quality [26]. Following the 3D GAN, the visual object network used the Wasserstein distance with the GP from the WGAN-GP [27,28]. In their rendering research, they introduced the PLATONIC GAN, which incorporates a rendering layer into the GAN architecture, thereby enabling the generation of 3D volumetric shapes from unstructured image collections [29]. Building on a Projective GAN, which includes 2D projections in 3D GAN training, a Multi-Projection GAN was also proposed for voxel-based generation [30,31].

### 2.3. Progressive Neural Networks

Progressive learning is a machine learning approach in which learning occurs incrementally by expanding the data and updating the model in stages instead of using the entire training dataset at once to train a model (as in traditional methods) [32]. Starting with a small dataset, the model is trained, and new data are progressively added to update the model. This mitigates issues due to data scarcity and enhances performance as more data become available. Moreover, simple concepts or patterns are initially learned, and the model is subsequently updated with more complex concepts and additional data. This allows the model to grasp the basic features before learning more abstract and intricate features for overall improvement.

Progressive neural networks involve adding new modules to a network when presented with a new task. Each module uses the output of the previous module as the input and constructs predictions for the new task. This enables the utilization of independent modules for new tasks while maintaining the performance of previous tasks [33]. In the context of transfer learning, progressive learning methods propose fixing a transfer learning neural network and adding weights to new classes, thereby maintaining the performance of prior tasks while learning new tasks [34]. Few-shot learning within progressive learning stores information about previous tasks and uses a small number of samples to train new tasks. This approach effectively enables the learning of new tasks while still utilizing existing information regarding prior tasks [35].

## 3. Proposed Method

### 3.1. The Architecture of the VAE Cascade GAN

In general, GANs consist of two networks: a generator that attempts to deceive the discriminator using generated samples that approximate the real data distribution, and a discriminator that estimates the probability that a sample comes from the real data distribution. These two networks were simultaneously trained using a min–max game. GANs provide a robust solution to the unsupervised problem of model data generation. While GANs aim to generate new model data, VAEs go beyond this by learning implicit representations and modeling data distributions. Specifically, in the VAE, the dependency of the latent variable z on distribution x is probabilistic, whereas in GANs, it is deterministic.

Our approach robustly combines the core components of the VAE for image feature extraction with the latent space of the generator and decoder in the GAN. It integrates a progressive network method and a modified discriminator loss into a GAN, demonstrating its great potential for generating 3D models with improved quality and diverse variations. The modified discriminator loss adapts the Wasserstein distance, a metric that measures the distance between distributions, to enhance the stability and generated quality.

**Figure 1:**
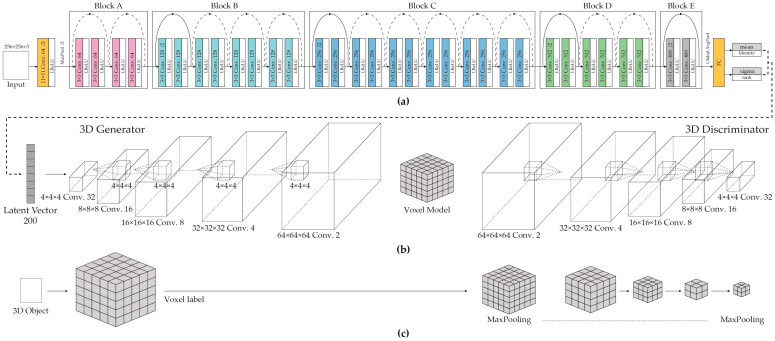
Architecture overview. The overall architecture comprises three components: (**a**) an improved ResNet34 Variational Autoencoder, (**b**) the mirrored architecture of the generator and discriminator Cascade GAN, and (**c**) a cascade discriminator for voxel labels.

As shown in Figure 1, the overall architecture comprises three stages: (a) a VAE for image features, (b) a progressively growing GAN, and (c) voxel labeling for 3D supervision. The PGGAN was trained by starting with a smaller voxel size and gradually adding layers to progressively increase the voxel size. Rather than training all the scales simultaneously, the focus was placed on the finer-scale details. The generator and discriminator networks were mirror images that grew concurrently. Transfer learning was performed as new layers were added. This approach allowed well-trained, smaller voxel layers to focus on the independent learning of the added layers. Our training began with a generator (G) and discriminator (D) for voxels with low-volume resolution. As training progressed, layers were progressively added to the generator and discriminator, increasing the spatial resolution of the generated voxels. All the existing layers were fine-tuned using the weights of the added layers.

### 3.2. Image-Based VAE

A VAE is a generative model that employs a probabilistic approach to represent the data features as latent variables and generates new data using these latent variables. The structure of a VAE consists of two main parts: an encoder and a generator. In a typical VAE setup, the encoder and generator are separate entities. The encoder encodes the input data into latent variables, and the generator uses these as inputs to generate new data.

The proposed approach combines the losses of the VAE and GAN to define the overall loss function. This involves combining the encoder and generator into a single network, implying that encoding and decoding (generation) are performed simultaneously within one network. This integrates the processes of generating latent variables and new data into a unified structure, thereby creating robust couplings. The reconstruction loss of the VAE and the generator and discriminator losses of the Wasserstein GAN (described in Section 3.3) were also considered. This approach emphasized the correlation between encoding the latent variables and decoding the results more effectively than a conventional VAE. Additionally, effective learning of latent variables and improved latent representations were observed, leading to the generation of data that closely resembled the original data.

Equation (3) represents the combined VAE loss, which combines the reconstruction loss and KL loss and is robustly integrated with the Wasserstein GAN, where x is the target sample, $\hat{x}$ is the sample generated from an encoded image and a random latent vector, μ and Σ are the means and variances produced by the encoder, respectively, and λ = 100. Equation (4) reflects the generator loss obtained using this approach:

(3) $${VAE}_{loss}={[\left||\hat{x}-x|\right|}_{2}]+KL[N(\mu ,\Sigma )||N(\left(0,\;I\right))]$$

(4) $$G_{loss}=D[G\left(z_{-}\right)]+\lambda {\left||\hat{x}-x|\right|}_{2}$$

### 3.3. Voxel-Based Cascade GAN

#### 3.3.1. Cascade GAN

In a traditional GAN, the structures of the generator and discriminator are fixed during training. Our approach introduces a technique called a progressive neural network to a 3D GAN, where the generator and discriminator are divided into multiple stages and progressively supervised. This method trains a GAN more effectively to generate complex 3D data. Progressive neural networks connect multiple GAN models to form a single large network. At each stage, the results generated using the model in the previous stage are used as inputs to train the model for the next stage. This enables information transfer between the generator and the discriminator, facilitating the transmission of features learned in the previous stages to generate large-scale 3D data.

Starting from the initial layers that generate simple 3D models, training progresses by accumulating layers and incrementally growing the model, thereby supervising the generator’s performance. As the generator’s ability to generate high-quality fake data improves, its structure expands, thus assisting it in learning the overall distribution of the data more effectively while progressively enhancing the discriminator’s evaluation of the generated data.

**Figure 2:**
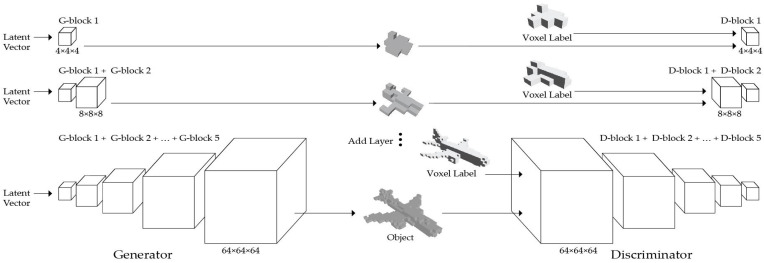
The mirrored cascade GAN architecture of the generator and discriminator with 3D voxel labels.

Figure 2 shows the expansion of the mirrored structure of the generator (G) and discriminator (D), doubling in volume while adding new layers. This illustrates a gradual transition from a 4 × 4 × 4 resolution to a 64 × 64 × 64 resolution. During the training of the discriminator, each voxel matches the current volume of the network. During the transition between volumes, the generator output interpolates between the two volume spaces, such as by combining the two volumes.

Overall, this technique aims to create a robust and progressively trained generator and discriminator within a 3D GAN framework.

Owing to its progressive training nature, our approach offers more stable learning and easier convergence at each stage. The initial stages have fewer classes and modes, increasing the stability in the generation of smaller sizes [36]. Additionally, we stabilized the progressive generation of the 64 × 64 × 64 resolution using the WGAN-GP loss [9].

Our cascade method starts with a low-resolution voxel size of 4 and trains progressively up to the high-resolution voxel sizes of 32 and 64. Other typical networks are processed with high-resolution 64-voxel size data from the beginning. Although the voxel size increases to 64 voxels by adding layers, the proposed method competes with networks that work with large voxels from the beginning. This means that lower voxel operations are relatively easy tasks and have lower computational costs than higher-resolution voxels.

Three main methods are used to avoid mode collapse. Two of them are the Wasserstein distance and gradient penalty described in related research. The last method starts by training the network with low-resolution voxels and gradually increasing them.

To enhance the quality of the small datasets, our method modifies the discriminator loss (L_D) of the Wasserstein GAN. The modified discriminator loss (L_D) is calculated using the latent vectors Z_i learned in the VAE by measuring the distance between the distribution of the generated data from the generator and the distribution of the labeled data x_i learned by the discriminator. Minimizing this distance during discriminator training encourages the generator to produce data similar to the learned data. The GP computes the gradients of the discriminator and utilizes them as constraints to stabilize both the generator and discriminator training. The weight λ of the GP is set considering stability during training. This approach allows the generator to learn a more accurate distribution and enhances the quality of the generated 3D voxels.

(5) $$D_{loss}=\frac{1}{n}\sum_{i=1}^{n}\left[D\left(x_{i}\right)-D\left\{G\left({z\_}_{i}\right)\right\}\right]+\lambda \frac{1}{m}\sum_{j=1}^{m}{\left({\left||\nabla_{{\hat{x}}_{j}}D({\hat{x}}_{j})|\right|}_{2}-1\right)}^{2}$$

Furthermore, our method involves adapting the architecture of a previously trained model and slightly fine-tuning its weights to suit new objectives. The layers added to the generator and discriminator initially share weights and undergo fine-tuning. The sublayers of the original model specialize in extracting high-level features, such as 3D volumes. The lower-level layers extract general and independent features while generating small volumes, whereas the higher-level layers extract specific and distinct features (shapes). These features are saved through fine-tuning for applications in different classes. By reusing weights from the original model and using fine-tuned weights for effective learning on small datasets, the proposed approach maintains a balance between generator and discriminator learning, prevents mode collapse, and facilitates the generation of larger-volume outputs while maintaining stable training.

#### 3.3.2. Voxel Labeling for Discriminators

Our method converts the original 3D model into a binary voxel format with a size of 128 × 128 × 128 pixels using the Binvox program (1.22). The original mesh model was transformed into a binary voxel format using tools such as Binvox (MagicaVoxel, Voxelizer, and MeshLab). Voxel labels for each layer were generated through 3D Max Pooling, which divides the input data into smaller regions and extracts the maximum value from each partitioned area to reduce the result. In this case, Max Pooling was applied multiple times to decrease the resolution. Additionally, when creating voxel labels, sum pooling and average pooling can be used by considering the surrounding information of individual voxels. The voxel labeling process is essential for training the Cascade GAN.

**Figure 3:**
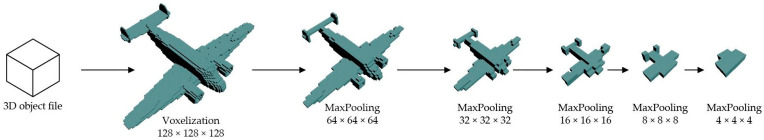
VAE Cascade GAN voxel labeling for discriminator learning.

Figure 3 illustrates the voxel labeling process used to train the discriminator. The voxel max pooling algorithm was utilized, and when reducing the voxel size through pooling, the number of voxels in the reduced cell was used as the input. For example, in a 2D space, four voxels are reduced to one, whereas in a 3D space, eight voxels are reduced to one, resulting in a maximum value of eight and a minimum value of one. In our experiments, when applying 2 × Max Pooling to the 128 × 128 × 128 data, the resolution was reduced to 64 × 64 × 64, and further applying 2 × Max Pooling reduced the resolution to 32 × 32 × 32. This process was repeated to achieve the desired resolution of 4 × 4 × 4. Additionally, sum pooling was applied to reflect the neighboring voxel information for labeling.

In Algorithm 1, the function Max Pooling 3D for voxel labeling takes the input data, pooling size, and stride as parameters. This algorithm calculates the output shape, iterates through the output shape to extract patches from the input data, computes the maximum value within each patch, and stores the results in the pooled data array.
**Algorithm 1.** Max Pooling 3D for voxel labeling.function Max Pooling 3D (input data, pooling size, stride):   input shape = shape of input data   output shape = ((input shape [0] − pool size)/stride + 1,           (input shape [1] − pool size)/stride + 1,           (input shape [2] − pool size)/stride + 1)   pooled data = new array with shape output shape   for i from 0 to output shape [0]:   for j from 0 to output shape [1]:    for k from 0 to output shape [2]:       start i = i × stride, start j = j × stride, start k = k × stride       end i = start i + pool size, end j = start j + pool size, end k = start k + pool size       patch = input data [start i: end i, start j: end j, start k: end k]       pooled data [i, j, k] = maximum value in patch   return pooled data

## 4. Experimental Evaluation

### 4.1. Experimental Setup

We experimented with three networks. These three networks were the proposed VAE Cascade GAN, the proposed ResNet VAE-WGAN, and the existing 3D-VAE-IWGAN.

The proposed VAE Cascade GAN was trained and optimized separately for the encoder, generator, and discriminator using the Adam optimizer. The learning rate for the encoder, generator, and discriminator was set to 0.0001. A batch size of 16 was used. This network converges at 1500 epochs, so the epoch size was set to 1500. The other two networks were trained and optimized separately, similar to the VAE Cascade GAN, and utilized the Adam optimizer with a learning rate of 0.0001. The batch sizes of the ResNet VAE-WGAN and 3D-VAE-IWGAN were configured to 64. The ResNet VAE-WGAN and 3D-VAE-IWGAN converge at approximately 4000 epochs. Therefore, the epoch size of these two networks was set to 4000.

#### 4.1.1. Dataset

The ShapeNet dataset [37] consists of 3D models of various categories, such as cars, chairs, handbags, animals, and airplanes. This dataset serves as an ideal benchmark for tasks such as 3D object recognition, classification, generation, and transformation. To evaluate our method, we conducted experiments using three categories of images in the ShapeNet dataset: airplane, chair, and bed images. The proposed network and competing networks used the same training/validation test split for the ShapeNet dataset.

Initially, we transformed the 3D objects from ShapeNet into input images with their corresponding labels. The input images were generated using the 3D computer graphics software Blender (2.90.0), incorporating random poses, lighting, and distances overlaid with random textures. The rendered images were created at a resolution of 256 × 256, with 15 input images generated per object. Ground-truth labels in the form of binary voxels were derived using the Binvox program by converting the 3D objects into a 128 × 128 × 128 resolution. The transformed objects were reconstructed as NumPy arrays, and the ground-truth voxel labels for the discriminator of the Cascade GAN were sequentially developed with resolutions of 64 × 64 × 64, 32 × 32 × 32, 16 × 16 × 16, 8 × 8 × 8, and 4 × 4 × 4 using 3D max pooling. This has the effect of being extended by new labeling. Such extensions address the issue of improperly determined parameters and poor network generalization by more effectively utilizing existing data [38,39,40]. In this study, 100 of the 130 models in each class were used as training data, and the remaining 30 were used as test data.

#### 4.1.2. Network Structure and Training Details

1. The Proposed VAE Cascade GAN.

Encoder: The encoder network was deeply structured using ResNet’s residual blocks. Leaky ReLU activation functions were utilized for all the layers except the last layer, and the structure was adapted from the ResNet-34 network. The final layer was configured to output the mean and sigma values of a 200-dimensional latent vector using identity and hyperbolic tangent (Tanh) activation functions to perform the role of the VAE encoder. The network’s architecture began with a Conv2D layer with a kernel size of 11 × 11 and max pooling in the first layer, followed by 16 residual blocks. A fully connected layer was employed in the last layer. The filter numbers in the residual blocks were, sequentially, 64, 128, 256, 512, and 400, with two, four, six, three, and one block, respectively. The encoder weights were initialized and set to their initial weights whenever the voxel volume increased.

Decoder (generator) and discriminator: During the training process of our network, G and D blocks were progressively added to the generator and discriminator, respectively. The G blocks consisted of Conv3D transpose layers, batch normalization layers, and ReLU activation functions. The D blocks were composed of Conv3D layers and activation functions. Each progressive layer was set to observe 1500 epochs, forming convergence curves for the various error functions. When the G and D blocks were added, an additional 1500 epochs were each executed, resulting in a total of 7500 epochs of training.

The process began with a 4 × 4 × 4 resolution and progressed to a 64 × 64 × 64 resolution. The initial 4 × 4 × 4 resolution generation comprised a dense layer, a reshaping layer, and a ReLU activation function. It was then upsampled using G blocks and transformed into voxels in the final layer of the generator. Except for the last layer, which employs a Leaky ReLU activation function, all the activation functions in the generator use ReLU. In the discriminator, the first layer receives voxels as inputs and transforms them into feature volumes, which are subsequently reduced to a single value using D blocks and Conv3D layers. Leaky ReLU activation functions were used throughout the discriminator. Additionally, batch normalization layers were excluded.

Table 1 shows the VAE Cascade GAN architectures of the full-resolution generator and discriminator.

2. The Proposed ResNet VAE-WGAN.

The proposed method was compared with the classic 3D-VAE-IWGAN [24], which was trained using the Wasserstein loss and GP. To compare the VAE Cascade GAN to the methods with 3D-generated results, we also proposed a variant ResNet VAE-WGAN by adding a variant ResNet34 and an adversarial layer configuration to the VAE-IWGAN framework. The variant ResNet34 uses a skip connection to solve the gradient loss problem, making it easy to transfer information and modify the modular structure for application to a 3D GAN.

3. Compared network.

For the 3D-VAE-IWGAN, the authors’ configuration was as follows [24]. The 256 × 256 resolution input image to the generator was passed through the fully connected layer with 2048 nodes of the VAE encoder as a 200-dimensional latent vector, and the generator of the GAN was a 3D deconvolution of 4 with a kernel size of 4 × 4 × 4 and a stride length of 2. The discriminator, i.e., the deconvolutional layer, comprises a batch normalization layer and a ReLU activation layer, in addition to the final layer, following only the Tanh activation layer. The output of the constructor was a voxel of 32 × 32 × 32 resolution.

Table 2 shows the 3D-VAE-IWGAN architectures of the generator and discriminator. The GAN involves a high level of computational complexity. Computational costs are reduced by reducing the number of parameters [41,42]. The computational cost is greater when more parameters are used than when using the VAE Cascade GAN.

### 4.2. Evaluation

**Figure 4:**
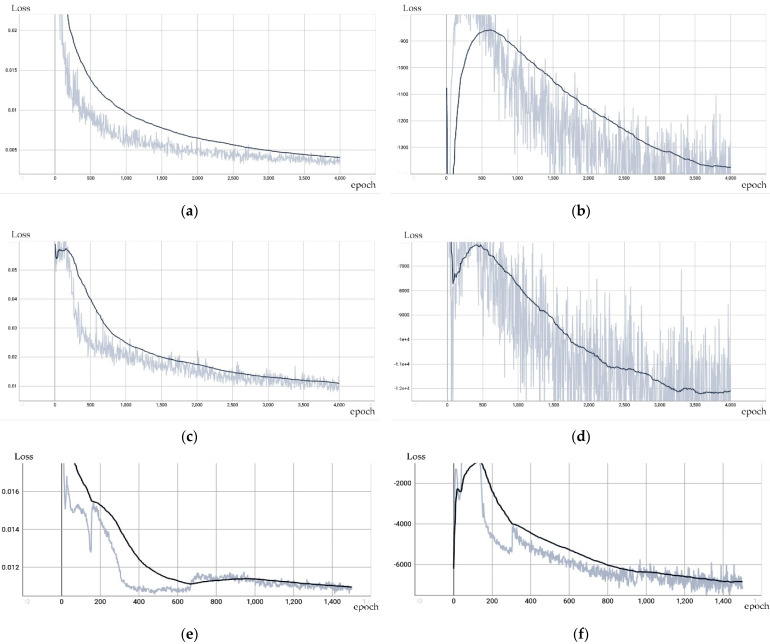
Loss value comparisons in the chair class. (**a**,**c**,**e**) Reconstruction loss. (**b**,**d**,**f**) Discriminator loss. (**a**,**b**) The loss of the 3D-VAE-IWGAN. (**c**,**d**) The loss of the ResNet VAE-WGAN. (**e**,**f**) The loss of the VAE Cascade GAN.

The stability of the proposed network was studied. As shown in Figure 4, the VAE Cascade GAN converges after approximately 1500 epochs, while the 3D-VAE-IWGAN and ResNet VAE-WGAN converge after approximately 4000 epochs. These experimental results show that our model is superior to the ResNet VAE-WGAN and 3D-VAE-IWGAN models in terms of convergence speed.

#### 4.2.1. Quantitative Evaluation

Our primary goal was to achieve stable learning and progressive growth to enable high output volumes. To compare the improved quality and diversity of the 3D volumes, we quantitatively evaluated our proposed modified ResNet VAE-WGAN, VAE Cascade GAN, and VAE-IWGAN [24] using established 3D evaluation metrics. This comparison provided a fair and quantitative basis for assessing both methods, considering the tasks performed using the 3D GANs.

Table 3 summarizes the results for the airplane category evaluated using the ShapeNet dataset. Our observations across various 3D volume evaluation metrics indicated the suitability of our approach.

Table 4 compares our proposed methods with existing approaches using 3D evaluation metrics, including the IoU, CD, RMSE, and FID for the three classes in the ShapeNet dataset. The improved stability and diversity of our method are confirmed. The FID is a metric used for evaluating the diversity of fake images [43,44].

#### 4.2.2. Qualitative Evaluation

**Figure 5:**
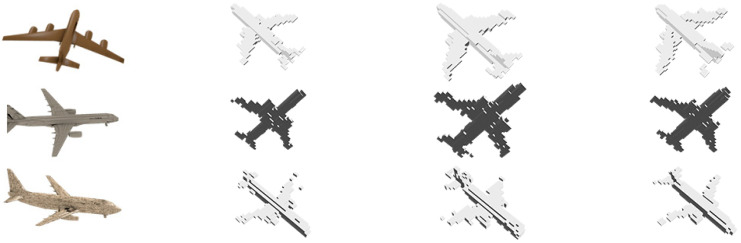

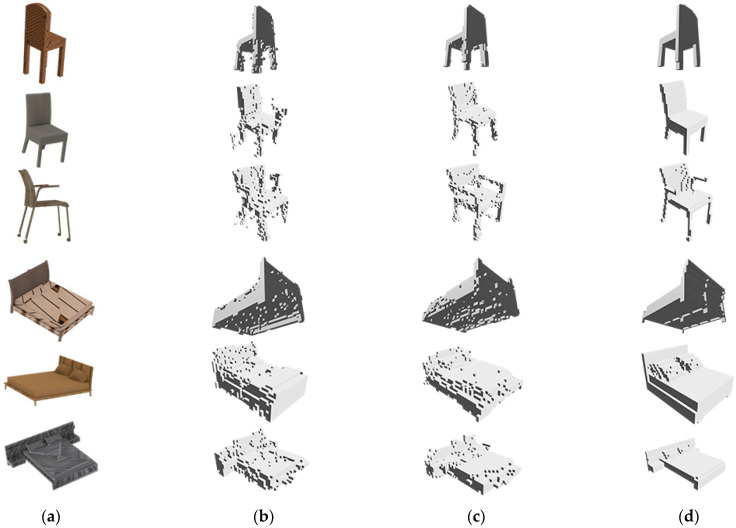
Visual results of the 3D reconstructions of three classes (airplane, chair, and bed) in different views. (**a**) Input image, (**b**) ResNet VAE-WGAN, (**c**) VAE Cascade GAN, and (**d**) voxel label.

Figure 5 shows the 3D reconstruction results for the three classes of proposed methods. The input images of the different views are shown in the first column. In the second column, the ResNet VAE-WGAN reconstructed a believable plane. With respect to the VAE Cascade GAN in the third column, we observed that the armrest of the chair was transforming into a natural shape. The fourth column shows the voxel label.

An important aspect is the growth of large resolutions at 64 × 64 × 64 during a stable learning process, and these 3D models not only yield improved quality in the input views but also in multiple views. The results for the chair category demonstrated the model’s ability to capture variations, ranging from straight-backed chairs to lounge chairs. Our approach shows visual competitiveness compared to classical 3D GAN systems and various prior approaches in terms of the evaluated classes.

**Figure 6:**
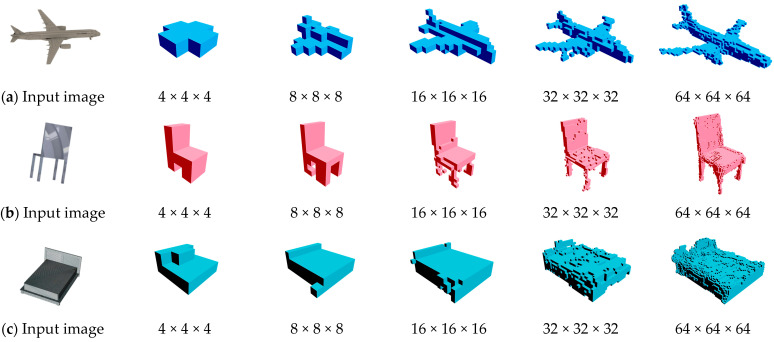
An example resolution reconstructed from a 256 × 256 image from the ShapeNet dataset. (**a**) An airplane, (**b**) a chair, and (**c**) a bed; level 4 × 4 × 4, 8 × 8 × 8, 16 × 16 ×16, 32 × 32 × 32, and 64 × 64 × 64 voxel grids.

Figure 6 shows the outcomes of the proposed approach, starting from a small resolution using 256 × 256 images and progressively increasing. It demonstrated stable and scalable reconstructions even at a voxel grid of 64 × 64 × 64.

**Figure 7:**
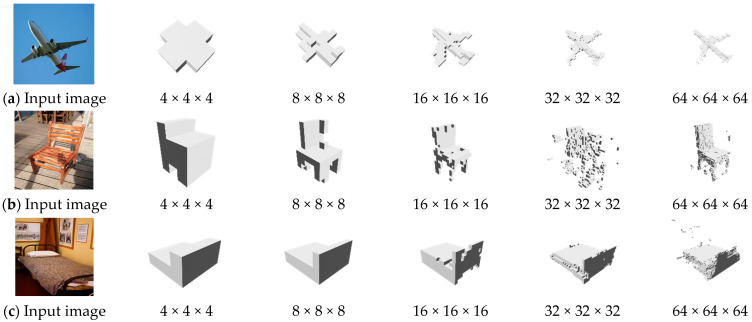
An example resolution reconstructed from a 256 × 256 real image: (**a**) an airplane, (**b**) a chair, and (**c**) a bed.

Figure 7 shows the outcomes of the proposed algorithm on 256 × 256 real images, which progressively increase in size. However, we identified certain quality issues in the results generated by some layers during the quality evaluation process.

## 5. Ablation Studies

By conducting an ablation study on the shape dataset, we investigated the contributions of the proposed cascade design and the discriminator’s objective function. We generated reconstructed volumes using the proposed Cascade GAN model with both the general D[G(z)] and the modified D[G(z_)] models.

Table 5 summarizes the quantitative results. Quantitative experiments were performed to validate the effectiveness of the modified D-Loss applied to the Cascade GAN. The modified D-Loss improved the majority of 3D evaluation metric values and consistently yielded stable results while progressively generating larger volumes in the initial stages. Additionally, we evaluated the results of constructing 3D shapes of objects found in images by converting them into images using the Fréchet Inception Distance (FID). This analysis highlights the significance of utilizing the general and modified D-Loss in our method.

Table 6 provides a summary of the evaluation of the Cascade GAN at a resolution of 64 × 64 × 64 voxels. The results in Table 6 show stable metrics, albeit with lower performance than for the the 32 × 32 × 32 voxel evaluation.

## 6. Conclusions

We propose a stable and scalable approach called the VAE Cascade GAN to progressively generate 3D models via a voxel-based approach to understand detailed voxel-based 3D shapes and discover enhanced 3D structures. Our approach efficiently utilizes small amounts of data and emphasizes stable training and the generation of high-quality and detailed 3D models. An effective integration of the VAE and GAN is achieved by designing modified objective functions for the VAE that learn meaningful image features during the reconstruction phase and for the progressively growing generator and discriminator of the GAN.

The generator and discriminator were constructed as 3D-mirrored structures by adding progressively larger layers as the training progressed to facilitate the stable expansion of 3D objects generated by the GAN. This approach entails the generator and discriminator, starting with small voxel sizes; as new layers are added, the discriminator utilizes labels to create a broader voxel space. Our method not only enhances the convergence speed but also improves the quality of the generated 3D models through stable growth.

We comprehensively evaluated the voxel quality and diversity, yielding results comparable to those of existing GAN-based methods. The generated 3D models excel in terms of their visual quality and detailed representation, rendering them suitable for various fields, such as virtual reality, gaming, and the metaverse. Our research focused on synthetic images in simulated scenarios. Real-scenario topics are only future tasks.

In future research, we aim to extend our approach to include texture mapping, volume layer separation, low computational costs, color mapping, transparency and blending, voxel-based rendering, and other techniques. This involves learning and assigning color information to each voxel as well as structuring the interior of the volume to provide better visual representations.

## Figures and Tables

**Table 1:** Generator and discriminator configurations used with the ShapeNet dataset to generate 64 × 64 × 64 voxels.

Generator	Act.	Output Shape	Params	Discriminator	Act.	Output Shape	Params
Input vector	-	200	-	Input Voxel	-	64 × 64 × 64 × 1	-
Dense	Identity	2048	411,648	Conv3D 1 × 1 × 1	LReLU	64 × 64 × 64 × 2	4
Reshape	-	4 × 4 × 4 × 32	-	D block	LReLU	32 × 32 × 32 × 4	516
BatchNorm	ReLU	4 × 4 × 4 × 32	128	D block	LReLU	16 × 16 × 16 × 8	2056
G block	ReLU	8 × 8 × 8 × 16	13,904	D block	LReLU	8 × 8 × 8 × 16	8206
G block	ReLU	16 × 16 × 16 × 8	3496	D block	LReLU	4 × 4 × 4 × 32	32,800
G block	ReLU	32 × 32 × 32 × 4	1105	Conv3D 4 × 4 × 4	LReLU	2 × 2 × 2 × 64	131,136
G block	ReLU	64 × 64 × 64 × 2	560	Conv3D 4 × 4 × 4	LReLU	1 × 1 × 1 × 128	524,416
Conv3D Trans	LReLU	64 × 64 × 64 × 1	5	Conv3D 4 × 4 × 4	LReLU	1 × 1 × 1 × 256	2,097,408
				Flatten	-	256	-
				Dense	-	1	257

**Table 2:** Generator and discriminator configurations used with the ShapeNet dataset to generate 32 × 32 × 32 voxels.

Generator	Act.	Output Shape	Params	Discriminator	Act.	Output Shape	Params
Input vector	-	200	-	Input voxel	-	32 × 32 × 32 × 1	-
Dense	ReLU	2048	411,648	Conv3D	LReLU	16 × 16 × 16 × 32	2080
Reshape	-	4 × 4 × 4 × 32	-	Conv3D	LReLU	8 × 8 × 8 × 64	131,136
BatchNorm	ReLU	4 × 4 × 4 × 32	512	Conv3D	LReLU	4 × 4 × 4 × 128	524,416
DeConv3D	-	8 × 8 × 8 × 16	2,097,280	Conv3D	LReLU	2 × 2 × 2 × 256	2,097,408
BatchNorm	ReLU	8 × 8 × 8 × 16	256	Flatten	-	2048	-
DeConv3D	-	16 × 16 × 16 × 8	524,352	Dense	Identity	1	2049
BatchNorm	ReLU	16 × 16 × 16 × 8	128				
DeConv3D	-	32 × 32 × 32 × 4	131,104				
BatchNorm	ReLU	32 × 32 × 32 × 4	64				
DeConv3D	Tanh	32 × 32 × 32 × 1	2049				

**Table 3:** Performance of different methods with regard to 3D volume evaluation metrics on ShapeNet (airplane). Root-mean-square error (RMSE), Intersection-over-Union (IoU), Chamfer Distance (CD), and Fréchet Inception Distance (FID) metric values were calculated. The lower the value, the better, except for the IoU.

Method	Voxel (32 × 32 × 32)			FID
	RMSE	IoU	CD	
3D-VAE-IWGAN [24]	0.1710	0.3329	0.4964	68.11
ResNet VAE-WGAN (ours)	0.1134	0.3942	0.0973	39.47
VAE Cascade GAN (ours)	0.1058	0.4220	0.0170	34.74

**Table 4:** Comparisons between our proposed method and competing approaches using 3D evaluation metrics.

Method	3D-VAE-IWGAN [24]			ResNet VAE-WGAN (Ours)			VAE Cascade GAN (Ours)		
	Chair	Bed	Average	Chair	Bed	Average	Chair	Bed	Average
RMSE	0.2460	0.3789	0.31245	0.2099	0.3420	0.27595	0.2449	0.2153	0.2301
IoU	0.2580	0.2254	0.2417	0.3040	0.3351	0.31955	0.3331	0.3394	0.33625
CD	0.2753	0.6282	0.4517	0.0303	0.0974	0.06385	0.0595	0.0410	0.05025
FID	159.55	178.96	169.255	137.00	137.56	137.28	120.54	96.02	108.28

**Table 5:** Performance of cascade layer volume generation of general D[G(z)] and proposed D[G(z_)]. The RMSE, IoU, CD, and FID on ShapeNet (airplane, chair, and bed). Lower values indicate better performances, except for IoU.

Ours		RMSE				IoU				CD				FID
		32	16	8	4	32	16	8	4	32	16	8	4	
Airplane	D[G(z)]	0.11	0.13	0.15	0.15	0.42	0.55	0.64	0.84	0.017	0.020	0.017	0.015	34.74
	D[G(z_)]	0.11	0.14	0.16	0.16	0.40	0.54	0.62	0.85	0.014	0.029	0.018	0.015	29.14
Chair	D[G(z)]	0.24	0.23	0.24	0.26	0.33	0.44	0.6	0.73	0.059	0.045	0.029	0.061	120.54
	D[G(z_)]	0.23	0.25	0.25	0.27	0.35	0.45	0.61	0.72	0.045	0.035	0.032	0.06	84.46
Bed	D[G(z)]	0.21	0.23	0.24	0.26	0.33	0.44	0.61	0.73	0.041	0.038	0.031	0.059	96.02
	D[G(z_)]	0.17	0.15	0.12	0.12	0.39	0.45	0.60	0.72	0.071	0.065	0.031	0.023	117.09

**Table 6:** Performance of the 64 × 64 × 64 voxel generation of the Cascade GAN. The RMSE, IoU, CD, and FID on ShapeNet (airplane, chair, and bed).

Method	Voxel (64 × 64 × 64)			FID
	RMSE	IoU	CD	
Plane	0.1002	0.3550	0.0121	71.93
Chair	0.1960	0.2829	0.0579	148.37
Bed	0.3489	0.2818	0.2918	214.10

## Data Availability

The code and dataset will be made available upon request from the first author’s email, with appropriate justification. The public site for each dataset is as follows: ShapeNet Dataset: https://shapenet.org/ (accessed on 15 August 2023).
